# Real-time imaging and analysis of differences in cadmium dynamics in rice cultivars (*Oryza sativa*) using positron-emitting^107^Cd tracer

**DOI:** 10.1186/1471-2229-11-172

**Published:** 2011-11-29

**Authors:** Satoru Ishikawa, Nobuo Suzui, Sayuri Ito-Tanabata, Satomi Ishii, Masato Igura, Tadashi Abe, Masato Kuramata, Naoki Kawachi, Shu Fujimaki

**Affiliations:** 1 Soil Environment Division, National Institute for Agro-Environmental Sciences, 3-1-3 Kannondai, Tsukuba, Ibaraki 305-8604, Japan; 2 Radiotracer Imaging Group, Medical and Biotechnological Application Division, Quantum Beam Science Directorate, Japan Atomic Energy Agency, Watanuki 1233 Takasaki, Gunma 370-1292, Japan; 3 Agricultural Research Institute, Ibaraki Agricultural Center, Kamikuniicho 3402, Mito, Ibaraki 311-4203, Japan

## Abstract

**Background:**

Rice is a major source of dietary intake of cadmium (Cd) for populations that consume rice as a staple food. Understanding how Cd is transported into grains through the whole plant body is necessary for reducing rice Cd concentrations to the lowest levels possible, to reduce the associated health risks. In this study, we have visualized and quantitatively analysed the real-time Cd dynamics from roots to grains in typical rice cultivars that differed in grain Cd concentrations. We used positron-emitting^107^Cd tracer and an innovative imaging technique, the positron-emitting tracer imaging system (PETIS). In particular, a new method for direct and real-time visualization of the Cd uptake by the roots in the culture was first realized in this work.

**Results:**

Imaging and quantitative analyses revealed the different patterns in time-varying curves of Cd amounts in the roots of rice cultivars tested. Three low-Cd accumulating cultivars (*japonica *type) showed rapid saturation curves, whereas three high-Cd accumulating cultivars (*indica *type) were characterized by curves with a peak within 30 min after^107^Cd supplementation, and a subsequent steep decrease resulting in maintenance of lower Cd concentrations in their roots. This difference in Cd dynamics may be attributable to OsHMA3 transporter protein, which was recently shown to be involved in Cd storage in root vacuoles and not functional in the high-Cd accumulating cultivars. Moreover, the PETIS analyses revealed that the high-Cd accumulating cultivars were characterized by rapid and abundant Cd transfer to the shoots from the roots, a faster transport velocity of Cd to the panicles, and Cd accumulation at high levels in their panicles, passing through the nodal portions of the stems where the highest Cd intensities were observed.

**Conclusions:**

This is the first successful visualization and quantification of the differences in whole-body Cd transport from the roots to the grains of intact plants within rice cultivars that differ in grain Cd concentrations, by using PETIS, a real-time imaging method.

## Background

Cadmium (Cd) has an important impact on agriculture, as the excessive consumption of Cd from contaminated food crops can lead to toxicity in humans. High-dose Cd exposure is particularly toxic to the kidney and leads to renal proximal tubular dysfunction [1]. In Japan, *itai-itai *disease (renal osteomalacia), which is characterized by complaints of spinal and leg bone pain, was recognized as a type of chronic toxicity induced by excess Cd contamination of drinking water and cereals (mainly rice). Since then, the contamination of rice by Cd has been monitored to prevent it from being distributed to consumers in Japan, in accordance with the Food Sanitation Act established in 1969 in Japan. Nevertheless, the Cd contamination of rice is still a serious threat to Japanese people and other populations in the world that consume rice as a staple food, because rice is a major source of dietary intake of Cd. Understanding how Cd is taken up by rice roots and subsequently transported to rice grains is necessary for reducing Cd concentrations in rice as much as possible, thus diminishing the risk that Cd poses to human health.

Plant roots are the first entry point for Cd uptake from soil solutions, and the transport processes of Cd into the roots have been well reviewed from the viewpoints of physiological and genetic studies [2]. A dose-dependent process exhibiting saturable kinetics has been shown in the roots of several graminaceous crops, including rice [3-5]. The saturable characteristics of Cd uptake could be controlled by a carrier-mediated system, and genetic studies in rice have indicated that the iron (Fe) transporters OsIRT1 and OsIRT2 and the zinc (Zn) transporter OsZIP1 can mediate Cd uptake by roots [6,7]. Once Cd enters into the root cells, its movement through the root symplasm to the xylem can be restricted by its sequestration in the vacuoles [8]. In tandem, apoplastic movement of Cd to the xylem can also be restricted by development of the endodermal suberin lamellae in the roots exposed to Cd [2]. Recently, it has been found that among rice cultivars varying in grain Cd concentrations, the differences in root-to-shoot Cd translocation rates via the xylem are affected by the P_1B_-ATPase transporter OsHMA3, which is involved in Cd sequestration in root vacuoles [9,10]. Xylem loading of Cd has been shown to be mediated by AtHMA2 and AtHMA4 in *Arabidopsis thaliana *[11,12]. In rice, functional assays by heterologous expression of *OsHMA2 *in yeast have suggested that this gene is a good candidate for the control of Cd xylem loading in rice [8]. The process of Cd unloading from the phloem is also recognized as a key factor for determining Cd levels in grains, because Cd moves to developing grains via the phloem [13,14]. Tanaka et al. [15] estimated that 91-100% of Cd in rice grains was deposited from the phloem when rice plants were treated with a relatively high Cd level with 1 μM Cd in hydroponics. Using an insect-laser method, Kato et al. [16] collected the phloem sap from the sheaths of the most expanded leaves of three rice cultivars differing in grain Cd concentrations, and found that the Cd concentrations of the phloem sap from these cultivars correlated well with their grain Cd concentrations. As described above, chemical and genetic analyses have provided many suggestions for every process in Cd transport in plants. Now, comprehensive information provided by whole-body and real-time observation of Cd movement in intact plants during vegetative and reproductive stages are needed for understanding the total plant system that leads to the difference of Cd concentrations between various cultivars.

In general, radioisotope tracers are useful tools for analysing the spatial distribution or temporal change in the amount of a substance in the plant body.^109^Cd has been widely used to visualize Cd distribution within plant tissues [17,18]. For example, Chino [17] observed that most Cd accumulated in the roots after isotope Cd (^109^Cd and^115m^Cd) supplementation at the early ripening stage, and lesser amounts of Cd were distributed to grains, whereas the lowest levels of Cd were present in the leaves. However, only the static distribution of Cd at a given moment can be obtained by autoradiography. In recent years, the positron-emitting tracer imaging system (PETIS) has been employed to study various physiological functions in intact, living plants [19,20]. This system enables not only monitoring of the real-time movement of the tracer in living plants as a video camera might, but also quantitative analyses of the movement of the substance of interest by freely selecting a region of interest (ROI) on the image data obtained. By applying this system to several graminaceous crops, the uptake and translocation of metals was investigated using positron-emitting tracers^52^Fe [21],^52^Mn [22], and^62^Zn [23]. Recently, Fujimaki et al. [24] established a real-time imaging system for Cd using positron-emitting^107^Cd tracer and PETIS. The movement of Cd in the aerial part of rice (cultivar Nipponbare) in the vegetative and reproductive stages was captured as serial images, and various parameters (e.g. transport velocity in the shoot) were analysed quantitatively. However, a method for direct imaging of the underground parts, which should provide valuable information about the root uptake, remained to be developed because of interference by the highly radioactive culture solution.

In this study, we employed PETIS in our two objectives: to realize direct observation of Cd uptake by the roots in the culture solution, and to characterize clearly the differences in Cd dynamics from the culture to the grains between the high- and low-Cd accumulating cultivars.

## Results

### Root^107^Cd uptake in different rice cultivars

**Figure 1:**
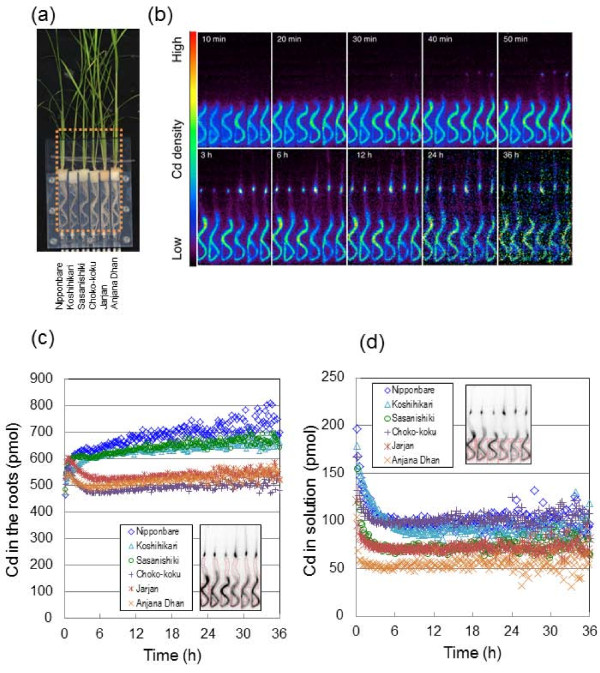
**Imaging and analysis of^107^Cd uptake by the roots of rice cultivars (vegetative stage)**. (a) Photograph of test plants. The large dotted rectangle indicates the FOV of PETIS. Nipponbare, Koshihikari, and Sasanishiki are of the *japonica *type, showing low Cd-accumulating cultivars. Choko-koku, Jarjan, and Anjana Dhan are of the *indica *type, showing high Cd-accumulating cultivars. (b) Serial images of Cd movement (0-36 h). (c) Time courses of Cd amounts in the roots surrounded by red lines in the black and white photograph. (d) Time course of Cd amounts in culture solution surrounded by red line. Cd in the roots (pmol) and Cd in solution (pmol) in Figure 1 indicate the sums of radioactive^107^Cd and nonradioactive Cd.

Figure 1 shows the imaging and analysis of Cd uptake by the roots among rice cultivars at the vegetative stage. The PETIS detectors were focused on the roots to monitor their^107^Cd dynamics (Figure 1a); data from the ROI of the roots were extracted for the quantitative analyses; and a time-course curve of Cd accumulation within the ROI was shown as the amounts of total Cd (pmol), consisting of the sums of radioactive and nonradioactive Cd (Figure 1c). An animation film of real-time Cd dynamics in the roots is available (Additional file 1). Serial images of root Cd distributions were obtained for 36 h (Figure 1b). Radical Cd uptake by roots was observed just after the^107^Cd was supplied (Figure 1b and 1c), irrespective of the cultivar types. This kinetics may reflect the binding of Cd within the apoplastic spaces of the root cell wall and the subsequent absorption via the plasma membrane into the cytoplasm, as seen in the root uptake patterns of divalent and trivalent cations [25]. In the three *indica *rice cultivars (Choko-koku, Jarjan, Anjana Dhan), which were classified as having markedly high Cd concentrations in their grains and shoots (herein collectively referred to as "high-Cd *indica *cultivars"), the amounts of Cd in the roots peaked within 30 min of exposure to^107^Cd, and the subsequent decreases in Cd were monitored until the 5 h point (Figure 1c). For the *japonica *rice cultivars (Nipponbare, Koshihikari, Sasanishiki) with lower Cd concentrations in their grains and shoots (herein collectively referred to as "low-Cd *japonica *cultivars"), the amounts of Cd in the Nipponbare and Sasanishiki roots plateaued or increased slightly after peaking at approximately 1 h. A delayed Cd peak was observed in the Koshihikari roots. In this study,^107^Cd was supplied only at the beginning of the imaging, and almost all of the^107^Cd in the culture solution was absorbed by the roots within approximately 5 h in all cultivars (Figure 1d). Therefore, the plateau observed in Figure 1c shows immobilization of Cd in the roots but not constant flow of Cd from the culture solution, and thus this shows that the low-Cd *japonica *cultivars have a greater ability to retain Cd in the root tissue compared with the high-Cd *indica *cultivars.

### Imaging of^107^Cd transfer to shoots in different rice cultivars

**Figure 2:**
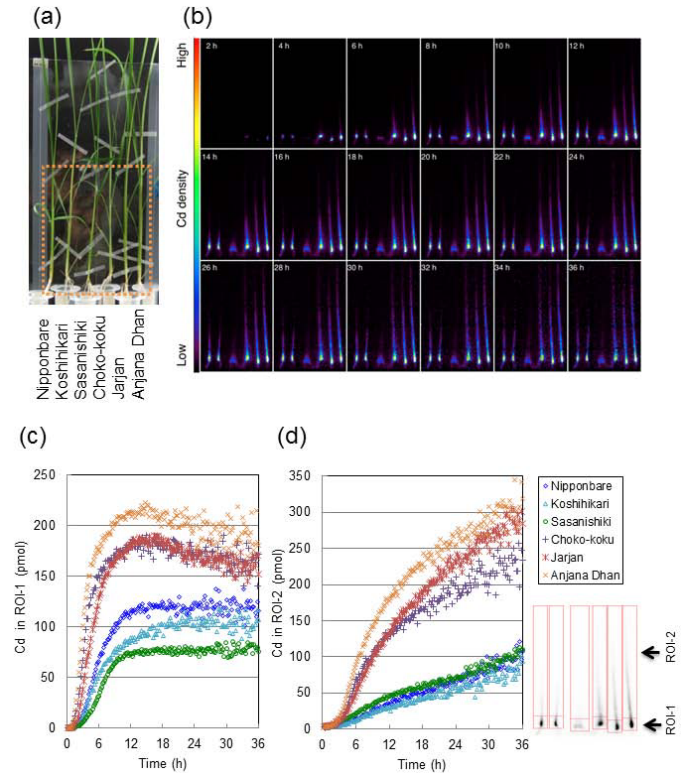
**Imaging and analysis of^107^Cd transport into shoots of six rice cultivars (vegetative stage)**. (a) Photograph of test plants. The large dotted rectangle indicates the FOV of PETIS. (b) Serial images of Cd movement (0-36 h). (c) Time course of Cd amounts in ROI-1 (shoot bases). (d) Time course of Cd amounts in ROI-2 (leaf sheaths and leaf blades). The relevant portion of each ROI is surrounded by red lines in the black and white photograph. Cd in ROI-1(pmol) and Cd in ROI-2(pmol) in Figure 2 indicate the sums of radioactive^107^Cd and nonradioactive Cd.

Figure 2 shows the imaging and analysis of Cd transport into the shoots of the six rice cultivars in the vegetative stage. The field of view (FOV) was focused on the shoots (Figure 2a), and serial images of Cd movement in each cultivar were monitored for 36 h (Figure 2b). An animation of Cd dynamics is displayed in Additional file 2. Cd first appeared and started to accumulate in the lower parts of the stems (shoot bases), or non-elongated stem part [26], showing intensive^107^Cd signals for all cultivars. The time-course curves of Cd amounts in ROI-1 (shoot base) and ROI-2 (leaf sheaths and leaf blades) are shown in Figure 2c and 2d, respectively. The Cd in ROI-1 began to accumulate within 1 h of^107^Cd supplementation and increased dramatically up to 10 h, particularly for the high-Cd *indica *cultivars. The amounts of Cd in ROI-1 were significantly higher in the high-Cd *indica *cultivars than in the low-Cd *japonica *cultivars up to 36 h. After 10 h, the amounts of Cd reached plateaus for all cultivars, but slight decreases were found in the high-Cd *indica *cultivars. Unlike the accumulation patterns of Cd in ROI-1, the amounts of Cd in ROI-2 (leaf sheaths and leaf blades) continued to increase linearly until the end of the experiment. There was an approximately 3-fold difference in the amount of Cd between the high-Cd *indica *cultivars and the low-Cd *japonica *cultivars.

**Figure 3:**
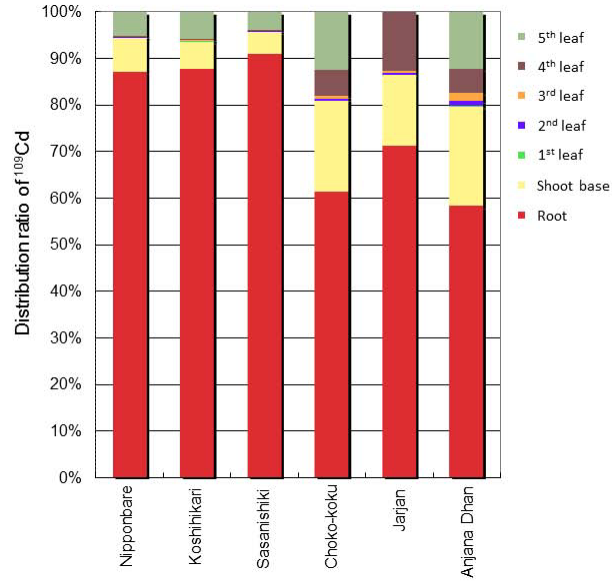
**Distribution ratios of Cd in the whole plants of rice cultivars at the vegetative stage**. After the PETIS experiment and the sufficient decay of^107^Cd within the test plants, autoradiography was carried out to obtain the static^109^Cd distribution in each plant part (Additional file 3), and the^109^Cd ratio was counted.

After the PETIS experiment, autoradiography was performed to obtain static distributions of Cd for each plant part at the vegetative stage (Additional file 3), and the distribution ratios of total Cd in their parts were calculated (Figure 3). Approximately 90% of the Cd absorbed by the *japonica *rice cultivars accumulated in their roots, whereas only 60-70% of the Cd in the *indica *rice cultivars was distributed in their roots. In the shoot parts, Cd accumulated at the shoot base in the highest proportions; this accounted for approximately 15-20% of the total Cd in the plant body for the high-Cd *indica *cultivars, whereas it was less than 10% for the low-Cd *japonica *cultivars. On the other hand, the proportions of Cd in the shoot base were approximately 50% of those in the total shoot and did not differ greatly between cultivars. In the leaves (leaf sheaths and leaf blades), Cd was mostly distributed in the younger leaves, that is, the 4th and 5th leaves, suggesting that Cd moves preferentially to new leaves after moving from the roots to the shoot bases.

### Imaging of^107^Cd transfer to panicle in different rice cultivars

**Figure 4:**
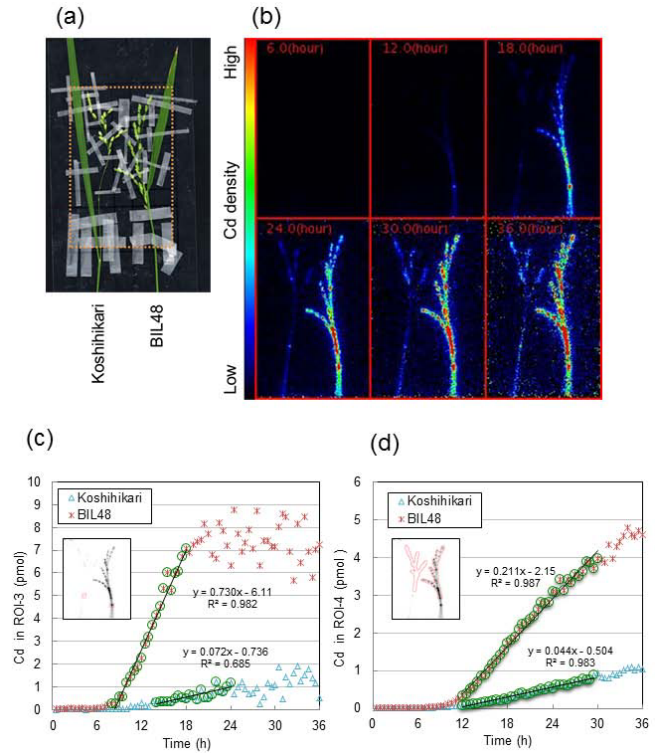
**Imaging and analysis of^107^Cd transport into the panicle for Koshihikari and BIL48**. BIL48 carries the QTL responsible for high Cd accumulation derived from Jarjan with the Koshihikari genetic background. (a) Photograph of test plants. The large dotted rectangle indicates the FOV of PETIS. (b) Serial images of Cd accumulation in the panicle (0-36 h). (c) Time course of Cd amounts in ROI-3 (neck nodes of panicles). (d) Time course of Cd amounts in ROI-4 (panicles). The Cd in ROI-4 refers to the Cd amount per glumous number. The relevant portion of each ROI is surrounded by red lines in the black and white photographs. Cd in ROI-3(pmol) and Cd in ROI-4(pmol) in Figure 4 indicate the sums of radioactive^107^Cd and nonradioactive Cd.

Figure 4 shows the imaging and quantitative analyses of Cd transport into the panicles of Koshihikari and backcross inbred line 48 (BIL48). BIL48 was used as a high-Cd accumulator, because it possesses a major quantitative trait locus (QTL) responsible for high Cd accumulation derived from Jarjan with the Koshihikari genetic background [27], and it shows synchronous panicle headings with Koshihikari by the short-day treatment. The FOV focused on the panicle (Figure 4a), and serial images of Cd movement into the panicle were monitored for 36 h (Figure 4b). The highest intensities of Cd, especially for BIL48, appeared in the culm, rachis, and neck node of the panicle within 12 h of^107^Cd supplementation. Cd showed a strong presence in the spikelets of BIL48 after 18 h, increasing steadily up to 36 h. In contrast,^107^Cd intensity in Koshihikari was lower throughout the experiment. Cd accumulation was not found in the flag leaf blade of either plant. Animation films of these images are also available (Additional file 4). The time course of Cd accumulation in ROI-3 (neck node of the panicle) and ROI-4 (panicle) are quantitatively analysed as shown in Figure 4c and 4d, respectively. The Cd accumulation in ROI-4 (Figure 4d) was calculated as the amount of Cd per one glume because the total numbers of glumes differ between Koshihikari and BIL48 (see Figure 4a). The initial increasing slopes (Figure 4c and 4d, circled plots) were fitted with lines depicting the kinetics of initial arrival of Cd in the respective ROI. The X-intercepts of the fitting lines were adopted as the arrival times of the theoretical "leading edge" of the Cd pulse, which are independent from the detection limit. Cd arrived in ROI-3 (Figure 4c) at 10.3 h and then accumulated at a gentle, linear slope up to 36 h in Koshihikari. In the Cd-accumulator BIL48, Cd arrived in ROI-3 8.4 h after supplementation and then increased at a steep, linear slope up to 18 h, finally reaching a plateau at approximately 7-8 pmol. In ROI-4 (Figure 4d), Cd arrived in Koshihikari at 11.4 h and then increased in concentration linearly at a gentle slope. For BIL48, Cd in ROI-4 arrived at 10.2 h and increased continuously at a steep slope up to 36 h. On the basis of the culm lengths (68.1 cm for Koshihikari and 67.4 cm for BIL48) and the estimated arrival times to the panicles (11.4 h for Koshihikari and 10.2 h for BIL48), the Cd transport velocities were calculated to be 6.0 cm h^-1 ^for Koshihikari and 6.6 cm h^-1 ^for BIL48. At the end of the PETIS experiment, the amount of Cd accumulated in ROI-4 was approximately 5-fold higher in BIL 48 than in Koshihikari.

**Figure 5:**
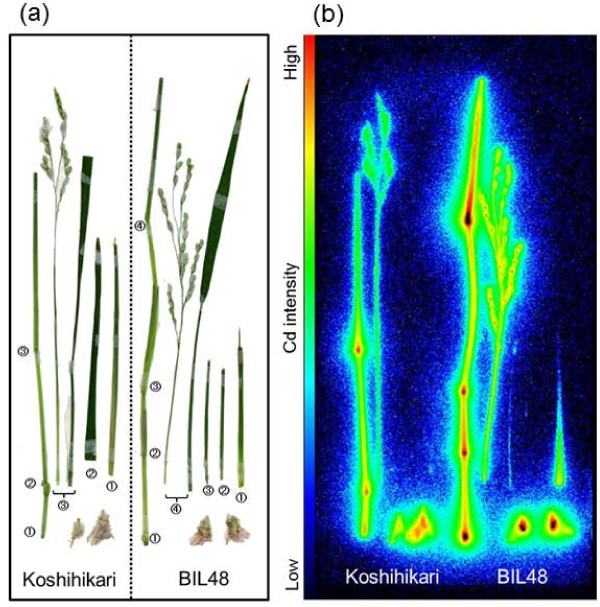
**Autoradiography of detached parts of shoots at grain-filling stage 36 h after supplementation with tracer**. After the PETIS experiment and the sufficient decay of^107^Cd within the test plants, autoradiography was carried out to obtain the static^109^Cd distribution in detached parts. (a) Photograph of test plants. (b) Autoradiograph of test plants. The same views are shown in (a) and (b). The pair of circled numbers indicates the nodal portions where the leaf was cut.

Both plants were subjected to autoradiography after the PETIS experiment (Figure 5a and 5b). A strong accumulation of Cd was observed in each node from the base to the top in both plants. In addition, Cd was present in the culms, rachises, and panicles in both plants. The Cd signals in these plant parts were remarkably stronger in BIL48 than in Koshihikari. The middle part of each glume in BIL48, where the ovary should be developing, showed a significantly strong Cd signal. In contrast, either no signal or a weak signal of Cd was detected in the leaf blades, even in the high-Cd accumulator BIL48.

## Discussion

### Improvement of the PETIS applicable to direct imaging of roots

It has long been considered technically impossible to observe the radiotracer-treated culture and the roots directly and simultaneously, because traditional imaging methods do not have a sufficiently broad range of detection that can accept such contrast. In this study, we principally improved three areas: 1) use of a root box with flat, shallow compartments, allowing detectors to focus on the roots; 2) use of a simple nutrient solution to avoid competition between Cd and other minerals at adsorptive sites in roots; and 3) ensuring application of adequate radioisotope activity for the quantitative measurements by taking into consideration the dynamic range of the PETIS. These technical improvements first enabled direct visualization of real-time Cd dynamics in the whole plant body, that is, from roots to grains.

We applied the improved system to analyse the time-varying distribution of Cd to characterize the differences in Cd dynamics in rice cultivars varying in grain Cd concentrations.

### Dynamic characterization of root Cd uptake and root-to-shoot translocation in rice cultivars differing in grain Cd concentration

The time courses of Cd amount in the root regions (Figure 1c) showed similar curves at the first 30 min as a rapid increase in all the cultivars tested, but were then followed by very different patterns between the cultivars. Three low-Cd *japonica *cultivars showed gentle saturation curves, whereas three high-Cd *indica *cultivars showed a drastic drop (Figure 1c). We consider that the curves in Figure 1c reflect the combination of the four successive functions of the root: adsorption to the outer root apoplast, absorption into the root symplast, retention within the cytoplasm or vacuole, and xylem loading. The very rapid increase at the first 30 min may reflect adsorption to the outer root apoplast, suggesting that this process was similar in all six cultivars. The subsequent drastic drop after 30 min in the high-Cd *indica *cultivars should be attributed to the simultaneous occurrence of two phenomena. One is depletion of Cd supply from the culture into the root as shown in Figure 1d, and the other is vigorous transfer of Cd from the root to the xylem. In contrast, the gentle saturation curves in the low-Cd *japonica *cultivars should indicate very low transfer from the root, because depletion of Cd supply from the culture was also the case in these cultivars (Figure 1d). Therefore, the different abilities between the low-Cd *japonica *cultivars and the high-Cd *indica *cultivars to transfer Cd from the root tissue into the xylem may have caused the most significant feature of Cd dynamics observed in the underground part.

This difference most probably depends on whether the rice plant inherently conserves the functional OsHMA3, which is a membrane transporter protein involved in Cd storage in root vacuoles. All high-Cd *indica *cultivars used in this study showed a loss of function of OsHMA3, resulting in failure to sequester Cd in their root vacuoles [9,10,28]. Our results indicate that loss of the sequestrating function of OsHMA3 into root vacuoles triggered transfer of Cd from the root tissue into the xylem within 30 min of contact between the root and Cd (Figure 1c). This result accords with a previous study that the radial transport of Cd in rice root from the culture to the xylem requires less than 10 min [24]. This transfer process was completed within 5 h (Figure 1c), which suggests that a concerted transport by absorption from the outer root apoplast into the symplast, and xylem loading from the symplast, takes place after very fast adsorption to the outer root apoplast. Moreover, the lack of drop after 30 min in the low-Cd *japonica *cultivars (Figure 1c) suggests that the sequestration function into root vacuoles is much more efficient than the xylem loading. These rapid dynamics seem to be specific to rice, because a previous study [29] showed that differences in root Cd concentrations between near-isogenic lines of durum wheat that differ in grain Cd concentrations were not observed until at least 4 days after Cd exposure. It should be noted that the kinetic curves in root Cd uptake were obtained with limited Cd (including^107^Cd) supply in this study, and this could be considered as a kind of pulse feed experiment. The curves obtained would naturally be different from those of roots with continuous Cd supply. The point is that the pulse feed experiments provide snapshots (temporal differentiation) of dynamics and the result with continuous feed could be described as their integration. In fact, the results from this study agreed well with our previous results obtained from the rice genotypes grown continuously in the Cd-polluted soil [5]; root Cd concentrations were higher in the low-Cd *japonica *cultivars than in the high-Cd *indica *cultivars.

In aerial parts,^107^Cd had a strong presence in the non-elongated stems at the shoot bases (Figure 2b) that contained densely packed nodes with complicated vascular bundle structures [30]. Other metals, such as Fe, Mn, and Zn, have also been shown to accumulate preferentially in this region in graminaceous crops [21-23], designated as the "traffic control centre" [31] or "discrimination centre" [32], and which plays important roles in distributing solutes taken up by the roots to each aerial tissue. The quantitative differences in Cd amounts in the shoot bases between low-Cd and high-Cd rice cultivars were apparent in the time course data (Figure 2c), and these were clearly in accord with the differing abilities of the cultivars to transfer Cd into the xylem. In addition, the slight decrease after the peak (at approximately 15 h) in the high-Cd cultivars (Figure 2c) indicates the relatively higher mobility of Cd from the shoot base (ROI-1) to the upper shoot parts (ROI-2). This tendency also seemed to be influenced by *OsHMA3 *gene expression in the shoot base, although the expression levels in the shoots are reported to be considerably lower than those in the roots [10]. The xylem parenchyma cells, having large vacuoles, are located in the centre of the enlarged xylem in the enlarged elliptical bundle of the node [26]. Xylem parenchyma and transfer cells play important roles in the selective absorption of solutes from the transpiration stream and their transport to the shoot apex [30,33]. If OsHMA3 function is defective in the xylem parenchyma cells in the high-Cd *indica *cultivars, Cd might move up to the upper leaf sheaths and leaf blades more easily through the transpiration stream, with reduced interception by the xylem parenchyma cells. However, in general, the proportions of Cd that finally accumulated in the shoot base after 36 h were approximately 50% of those in the total shoot, and did not differ greatly between the cultivars (Figure 3). This might suggest that the xylem unloading function was barely influenced by the genetic difference between the cultivars tested even though the Cd amounts loaded into the xylem were largely varied. Cd deposited temporarily in the shoot base seems to be translocated preferentially into the youngest developing leaves (Figure 3). The preferential translocation of Zn [34] and Fe [21] into the youngest leaves in graminaceous crops has also been reported. In a previous study, it was found that^52^Fe translocation to the youngest leaves of barley seedlings can be severely suppressed by a steam-girding treatment of the leaves, which inactivates phloem but not xylem transport, suggesting that Fe is mainly translocated to the youngest leaves via the phloem [21]. Fujimaki et al. [24] showed that Cd moved from the shoot base into the crown roots, which were split and kept away from direct contact with the Cd solution, suggesting that Cd was transferred from the xylem to the phloem at the nodes in the shoot base. These findings suggest that preferential and high Cd accumulation in the youngest leaves, especially for the high-Cd cultivars, could be partially explained by high levels of Cd in the phloem after the xylem-to-phloem transfer of Cd at the shoot base, where the high Cd signals were observed for the high-Cd cultivars.

### Dynamic characterization of Cd accumulation in panicles of rice cultivars that differ in grain Cd concentration

The Cd accumulation pattern of the neck node for the high-Cd accumulator BIL48 plants corresponded well to that of the node at the shoot base, showing the characteristic steep and linear increase, and subsequent plateau pattern of Cd accumulation (Figures 2c and 4c). Therefore, the neck node of the panicle may participate in the traffic control centre that distributes Cd to each spikelet. The linear accumulation pattern of Cd in the panicle was observed in both rice plants after^107^Cd reached the respective panicle, although the accumulated levels differed substantially between plants (Figure 4d). Fujimaki et al. [24] quantified the velocity of the long-distance transport of Cd through the shoot at the grain-filling stage to be 5.4 ± 0.4 cm h^-1 ^in the low-Cd cultivar Nipponbare. In this study, it was estimated to be 6.0 cm h^-1 ^for the low-Cd cultivar Koshihikari, and the value seemed to be similar. The transport velocity of Cd for the Cd accumulator BIL48 (6.6 cm h^-1^) was found to be slightly faster than that for Koshihikari. However, the differences in the Cd transport velocity between genotypes were likely to be small. Instead, a remarkable difference (approximately 5-fold) was observed in the slopes of Cd accumulation to panicles. Therefore, this result indicates that the differences in root Cd dynamics also influence the Cd concentration of the long-distance Cd transport to panicles in rice cultivars.

Interestingly, at 36 h no Cd was found to be distributed in the flag leaves of either plant in the PETIS experiment, in which^107^Cd was supplied to the genotypes with emerged ears (Figures 4b and 5). In contrast, significant Cd accumulation was seen in all nodes of the elongated stems of both plants, especially at the uppermost node I, which is connected to the flag leaf and panicle. Node I functions in the distribution of solutes from the roots to the flag leaf or panicle [26,33]. The autoradiography results suggest that the Cd at node I translocated preferentially to the developing panicle and not to the developed flag leaf, but the method by which node I determines the destination of Cd is unknown. Silicon transport to rice grains has been proposed to be involved in the inter-vascular transfer from the enlarged vascular bundles to the diffuse vascular bundles, passing through the xylem transfer cells present in the parenchyma cell bridge at node I, and a transporter related to inter-vascular transfer has been identified [35]. The diffuse vascular bundles of node I are assembled in internode I to form large vascular bundles that connect toward the panicle tissues [26,35]. Using a synchrotron micro X-ray fluorescence spectrometer and electron probe micro analyser, Cd was detected in the phloem of large vascular bundles at node I (Yamaguchi et al. 2011, submitted). In addition, it has been reported that the xylem-to-phloem transfer of Cd takes place in the nodes of rice [24], and the dominant route of Cd transport in brown rice is the phloem [15,16]. Our findings and these reports largely indicate that Cd passes through the phloem of the large vascular bundles in internode I after the xylem-to-phloem transfer at node I, and the Cd concentrations in the phloem may affect the genotypic differences in Cd accumulation in rice grains.

In paddy fields, rice is mostly grown under submerged conditions in which bioavailable Cd is limited because of the rise in soil pH and decrease in the redox potential. Midseason drainage in Japanese paddy fields is widely recommended at the vegetative stage to avoid the root rot induced by continuous soil reduction. In addition, early drainage after panicle emergence is often practised in paddy fields to facilitate machine harvesting. Thus, rice is not continuously exposed to high bioavailable Cd in the soil, and the PETIS data obtained by a limited Cd (including^107^Cd) supply might be a description of the Cd dynamics in rice at the vegetative and heading stages after water drainage in the paddy fields.

Thus, the PETIS is a very effective tool for comprehensively evaluating Cd dynamics from roots to grains, and for predicting the physiological processes of Cd transport in intact plants. The imaging and kinetics data have clearly demonstrated the differential Cd dynamics in the living plants of rice cultivars. The dynamics could be influenced by many physiological and biochemical steps, in which multiple genes controlling Cd dynamics are involved. For instance, using the various mapping populations, the major QTLs responsible for Cd accumulation in rice were detected on chromosomes 3, 4, 6, 7, 8, and 11[36-38], suggesting that the genotypic variation in Cd transport in rice is controlled by multiple genes. In this study, we happened to select three high-Cd *indica *cultivars that carry the non-functional alleles of OsHMA3, based on previously screened data relating to Cd accumulation in many rice cultivars[5]. In the near future, we intend to analyse the Cd dynamics in high-Cd cultivars carrying alterations in responsible genes other than OsHMA3. This experimental system would be appropriate for detailed functional analyses of the various genes responsible for Cd transport.

## Conclusions

Using the PETIS, we made the first direct observation of Cd uptake by the roots in the culture solution, characterized the successive transport processes in the root tissues, and described the differences in real-time Cd dynamics from the roots to the grains between the high- and low-Cd accumulating rice cultivars. The apparent differences were clearly shown as Cd retention in the roots, the rates of Cd translocation from the roots to the shoots, and the long-distance Cd transport to the panicles. Our studies have clearly connected the difference in gene function in the rice cultivars with *in vivo *movement of Cd from the culture through the root to the shoot in rice plants.

## Methods

### Plant materials

For the experiments conducted at the vegetative seedling stage, we used six rice cultivars (*Oryza sativa *L.) consisting of three *indica *rice cultivars (Choko-koku, Jarjan, Anjana Dhan) with markedly high Cd concentrations in their grains and shoots, and another three major *japonica *cultivars from Japan (Nipponbare, Koshihikari, Sasanishiki) with lower Cd concentrations in their grains and shoots [5]. Koshihikari and a BIL derived from Koshihikari and Jarjan (BIL48) were used for the experiments conducted at the grain-filling stage. BIL48 possesses a major QTL responsible for high Cd accumulation in shoots [27]. The seeds were soaked in deionized water for 2 days at 32°C and transferred to a nylon mesh floating on 20 L of a 1/2 strength Kimura B solution. The complete nutrition solution consisted of 0.36 mM (NH_4_)_2_SO_4_, 0.36 mM Ca (NO_3_)_2_·4H_2_O, 0.54 mM MgSO_4_·7H_2_O, 0.18 mM KNO_3_, 0.18 mM KH_2_PO_4_, 40 μM Fe(III)-EDTA, 18.8 μM H_3_BO_3_, 13.4 μM MnCl_2_·4H_2_O, 0.32 μM CuSO_4_·5H_2_O, 0.3 μM ZnSO_4_·4H_2_O, and 0.03 μM (NH_4_)_6_Mo_7_O_24_·4H_2_O. Kimura B solution has been widely used for growing rice plants [5]. The solution was replaced once a week, and the pH was adjusted to 5.2 every day. The seedlings of six cultivars were grown for 2-3 weeks in a greenhouse under natural sunlight and used for the vegetative stage experiments.

Three weeks after sowing, the seedlings of Koshihikari and BIL48 were transplanted to the full-strength Kimura B solution and grown to the heading stage in a growth chamber. The plants were exposed to a short-day treatment with an 8-hour photoperiod, day/night temperatures of 30°C/25°C, relative humidity of 70%, and light intensity of 400 μmol m^-2 ^s^-1 ^in order to synchronize the first-ear emergences of Koshihikari and BIL48. Koshihikari was examined at 9 days, and BIL48 at 5 days, after the first ear emergence for the grain-filling stage experiments.

### ^107^Cd tracer and PETIS imaging

The^107^Cd isotope was produced following the method of Fujimaki et al.[24]. Briefly, a silver foil was bombarded with a 17 MeV energetic proton beam at a current of 2 μA from a cyclotron at Takasaki Ion Accelerators for Advanced Radiation Application (Japan Atomic Energy Agency). The^107^Cd in the irradiated target was purified by an AgCl_2 _precipitation reaction after the addition of 2 M HCl. Finally, 7.6-60.2 MBq of^107^Cd was fed to each test plant depending on the experiments described below.

The PETIS imaging experiments were conducted following the method of Fujimaki et al. [24] with modifications to visualize the dynamics of Cd uptake by the root. First, an acrylic root box 187 mm (height) × 120 mm (width) × 10 mm (depth) was partitioned into six cells, each cell being 187 mm (height) × 17.5 mm (width) × 10 mm (depth). This box was devised to focus the detectors on the root surfaces in the radiotracer-treated culture solution, enabling observation of the multiple roots simultaneously. The root box consisted of two parts: an acrylic board with partitioned cells, and a flat acrylic plate for covering (Figure 1a). The lower leaf sheaths of the test plants were held with surgical tapes onto the board and the roots were placed in each cell compartment, supported by plastic sheets with small holes, and covered by the flat plate. The board and plate were completely sealed with screws and the culture solution was poured into each cell compartment. Second, to avoid competition between Cd and other minerals (e.g. Zn, Fe, and Mn) at adsorptive sites in the roots and so prevent a consequently low spatial resolution of Cd dynamics, the culture used for imaging was altered to a 0.5 mM CaCl_2 _solution instead of the full-strength nutrient solution used by Fujimaki et al. [24]. Finally, taking into consideration the wide dynamic range of the PETIS, we determined the amounts of radioisotope adequate for root imaging using the simple solution in the root box. These improvements enabled direct and simultaneous observation of the radiotracer-treated culture and the roots. The plants were acclimatized in a 0.5 mM CaCl_2 _solution (pH 5.2) for 24 h before the start of the^107^Cd supplementation experiment. The solution was continuously aerated, and the surface levels were set a few centimetres below the boundaries between the shoot bases and roots by automatically supplying fresh solution from the reservoir tank as the plants took up the water. Purified^107^Cd and nonradioactive Cd at concentrations of 0.1 μM were simultaneously supplied as carriers to the 0.5 mM CaCl_2 _solution in which the plants were grown. Plants were placed in the mid-plane between the two opposing detector pairs of the PETIS apparatus (a modified PPIS-4800; Hamamatsu Photonics, Hamamatsu, Japan). A pair of annihilation γ-rays emitted from the decaying positrons was detected simultaneously, and the emission point was then determined as the middle point of the two incident points. Repeated determinations of the emission points reconstructed a static image of the tracer distribution. One frame, which is the unit of time required to obtain one static image with sufficient quality, was set to 4 min, and 540 (36 h) frames were collected to yield serial time-course imaging. The detectors were set at the roots, non-elongated stem bases (shoot bases), and panicles to monitor the dynamics of Cd in each part. The typical size of the FOV in the detector head was 12 cm in width and 19 cm in height, and the spatial resolution was approximately 2 mm. All PETIS experiments were conducted in a growth chamber at 30°C and 70% humidity, with continuous light at a density of 400 μmol m^-2 ^s^-1^.

### Qualitative and quantitative analyses of PETIS data

To determine Cd dynamics in the plant body qualitatively and quantitatively, the dataset obtained from the PETIS apparatus was reconstructed using the NIH Image J 1.42 software (http://rsb.info.nih.gov/ij). Because the ROI can be selected freely from the image data using this software, the radioactivity of^107^Cd over time within each ROI was extracted from the data. A time-course curve of Cd accumulation within the ROI indicated the amounts of total Cd, consisting of the sums of radioactive and nonradioactive Cd. All PETIS experiments were conducted two or three times, and the representative data are shown in this paper.

### Autoradiography

In the production process of^107^Cd, gamma-ray-emitting^109^Cd was also produced at a minor ratio (approximately 1/3000). This isotope has a longer half-life (461 days) than^107^Cd (6.5 h), and it was absorbed by the plants during the PETIS experiments but not detected by the PETIS apparatus because it is not a positron emitter. After sufficient decay of^107^Cd within the test plants, they were separated into several parts and set on imaging plates (Fujifilm, Tokyo, Japan) in cassettes. After a few days of exposure, the imaging plates were scanned using a bio-imaging analyser (BAS-1500, Fujifilm, Tokyo, Japan) to obtain the autoradiographic images for examining^109^Cd distribution in the plant bodies. The Cd concentrations in each plant part were determined with a well-type gamma counter (ARC-7001; Aloka Co., Ltd., Tokyo, Japan).

## Authors' contributions

SI^1^, NS, and SF initiated and coordinated the study. SI^1^, MI, TA, and MK prepared the experimental plants and participated in the PETIS imaging. NS, SIT, SI^2^, NK, and SF produced the^107^Cd tracers and carried out the PETIS imaging. NS and SIT processed the imaging dataset obtained by the PETIS. SI^1 ^drafted the manuscript with the assistance of NS and SF. All authors discussed the results and commented on the draft manuscript, and read and approved the final manuscript.

## Supplementary Material

Additional file 1 **Animation film of^107^Cd dynamics in the roots of six rice cultivars at the vegetative stage**. Click here for file

Additional file 2 **Animation film of^107^Cd transport into shoots of six rice cultivars at the vegetative stage**. Click here for file

Additional file 3 **Autoradiography of detached parts of plants at the vegetative stage 36 h after Cd supplementation**. A, Nipponbare. B, Koshihikari. C, Sasanishiki. D, Choko-koku. E, Jarjan. F, Anjana Dhan. Click here for file

Additional file 4 **Animation film of^107^Cd accumulation in the panicles of Koshihikari and BIL48 at the grain-filling stage**. Click here for file
