# Supplementary figures and images for: Real-time imaging and analysis of differences in cadmium dynamics in rice cultivars (Oryza sativa) using positron-emitting107Cd tracer
Source: BMC Plant Biol. 2011 Nov 29;11:172. doi: 10.1186/1471-2229-11-172 (PMC3247196; doi:10.1186/1471-2229-11-172)

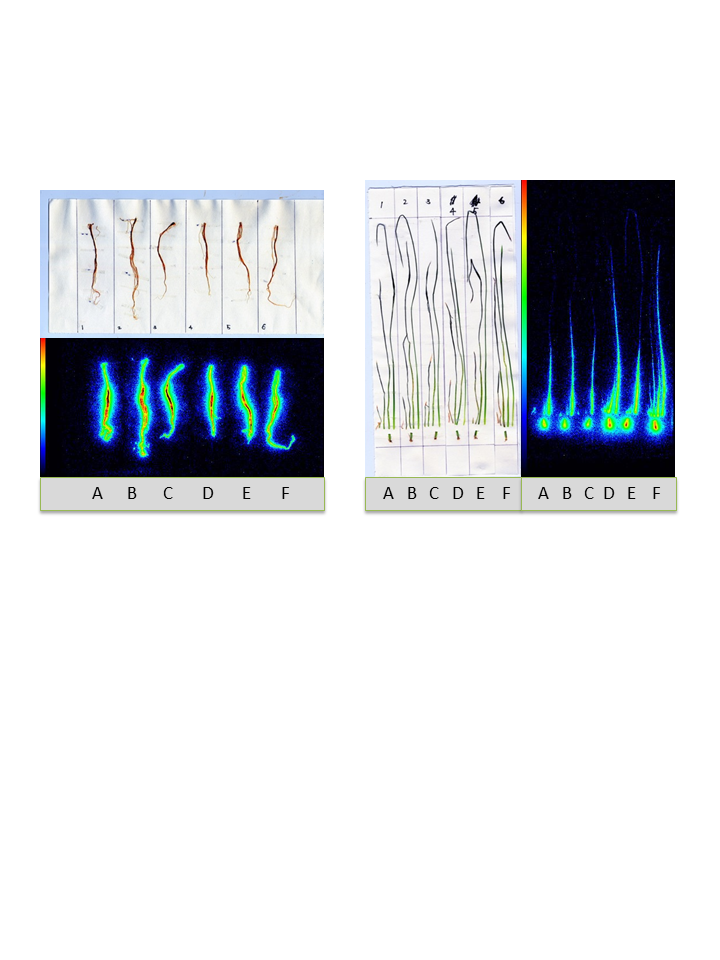

Supplement: Additional file 3 — Autoradiography of detached parts of plants at the vegetative stage 36 h after Cd supplementation. A, Nipponbare. B, Koshihikari. C, Sasanishiki. D, Choko-koku. E, Jarjan. F, Anjana Dhan. [file 1471-2229-11-172-S3.TIFF]
